# Dissecting cellular senescence and SASP in *Drosophila*

**DOI:** 10.1186/s41232-016-0031-4

**Published:** 2016-12-05

**Authors:** Takao Ito, Tatsushi Igaki

**Affiliations:** Laboratory of Genetics, Graduate School of Biostudies, Kyoto University, Yoshida-Konoecho-cho, Sakyo-ku, Kyoto, 606-8501, Japan

**Keywords:** Cell Cycle Arrest, Cellular Senescence, Senescent Cell, Hippo Pathway, Hippo Signaling

## Abstract

Cellular senescence can act as both tumor suppressor and tumor promoter depending on the cellular contexts. On one hand, premature senescence has been considered as an innate host defense mechanism against carcinogenesis in mammals. In response to various stresses including oxidative stress, DNA damage, and oncogenic stress, suffered cells undergo irreversible cell cycle arrest, leading to tumor suppression. On the other hand, recent studies in mammalian systems have revealed that senescent cells can drive oncogenesis by secreting diverse proteins such as inflammatory cytokines, matrix remodeling factors, and growth factors, the phenomenon called senescence-associated secretory phenotype (SASP). However, the mechanisms by which these contradictory effects regulate tumor growth and metastasis in vivo have been elusive. Here, we review the recent discovery of cellular senescence in *Drosophila* and the mechanisms underlying senescence-mediated tumor regulation dissected by *Drosophila* genetics.

## Background

Cellular senescence has been considered to be a major defense mechanism against carcinogenesis through the induction of stable cell cycle arrest [1–6]. Aberrant oncogene activation such as Ras activation causes various stresses including oxidative stress and DNA damage, thereby leading to the induction of premature senescence independently of telomere emersion [2, 3, 5–18]. This oncogene-induced senescence (OIS) can block malignant progression of precancerous lesions [5–7, 16]. However, recent studies have indicated that senescent cells can also contribute to tumor progression via the release of secretory components such as inflammatory cytokines, matrix remodeling factors, and growth factors, which is called the senescence-associated secretory phenotype (SASP) [19–22]. Thus, cellular senescence has not only negative effects but also positive effects on tumor development. Therefore, elucidation of how senescent cells drive both tumor suppression and tumor progression through cell–cell communications in vivo is essential if taking into account cellular senescence as a therapeutic target for cancer.

The genetic mosaic technique available in *Drosophila* is a powerful tool to study cell–cell communications in vivo [23, 24]. This technique allows us to analyze in vivo interactions between senescent cells and surrounding cells during tumor progression. In this review, we describe the recent identification of cellular senescence in *Drosophila*, as well as the recent advances in our understanding of the mechanisms by which senescent cells drive tumor progression via SASP in *Drosophila*.

## Cellular senescence and SASP in *Drosophila*

Since the first discovery by Hayflick and Moorhead in 1961 [25], cellular senescence has been widely studied in mammalian cells. Cellular senescence is known as a stepwise process from early senescence to full senescence [26–30]. In an early senescence state, senescent cells exhibit senescence-associated β-galactosidase (SA-β-gal) activity [31, 32], elevated expression of cyclin-dependent kinase (CDK) inhibitors such as p16 [12, 33, 34] and p21 [12, 35–37], reversible cell cycle arrest, senescence-associated heterochromatic foci (SAHF) [38–41], and cellular hypertrophy [31]. When matured to a full senescence state, senescent cells exhibit additional phenotypes including irreversible cell cycle arrest and SASP. Despite the extensive studies of cellular senescence in vertebrate models, there has been no evidence that cellular senescence also occurs in invertebrates.

**Fig. 1:**
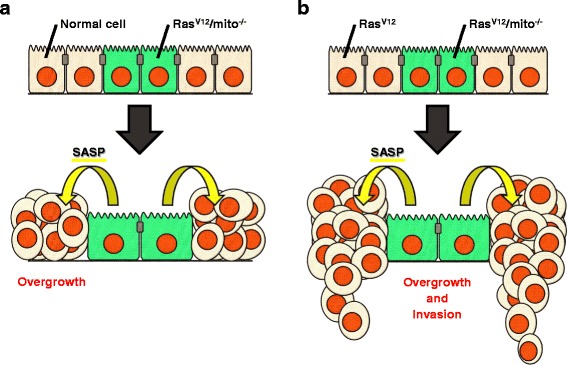
Senescent Ras^V12^/*mito* ^−/−^ cells trigger non-autonomous overgrowth of surrounding cells via SASP in *Drosophila*. **a** Ras^V12^/*mito* ^−/−^ cells induce non-autonomous overgrowth of surrounding normal cells. **b** Ras^V12^/*mito* ^−/−^ cells induce non-autonomous overgrowth and invasion of surrounding Ras^V12^ cells

Using *Drosophila* genetics, it has recently been shown that the state of full senescence can be induced by simultaneous activation of the Ras oncogene and mitochondrial dysfunction in *Drosophila* imaginal epithelium [42, 43]. Clones of cells with Ras activation and dysfunction of the mitochondrial electron transport chain (Ras^V12^/*mito* ^−/−^ clones), both of which are frequently observed in various types of human cancers [44–48], show elevated SA-β-gal activity, cell cycle arrest accompanied with upregulation of the Cdk inhibitor Dacapo (a *Drosophila* p21/p27 homologue), SAHF, and cellular hypertrophy [42]. In addition, Ras^V12^/*mito* ^−/−^ cells present SASP, as these cells excessively secrete the inflammatory cytokine Unpaired (Upd; a *Drosophila* interleukin 6 (IL-6) homologue [49]) and matrix metalloprotease 1 (Mmp1; the *Drosophila* secreted Mmp [50]), thereby causing non-autonomous overgrowth of neighboring cells (Fig. 1) [42, 43]. IL-6 and Mmp are known as SASP factors in mammals [21]. Intriguingly, clones of cells with Ras activation alone (Ras^V12^ clones) show elevated SA-β-gal activity, Dacapo upregulation, SAHF, and cellular hypertrophy but not cell cycle arrest and SASP [42]. Thus, Ras activation alone is insufficient for the induction of full senescence in *Drosophila* imaginal epithelium. Accordingly, mitochondrial dysfunction seems to be crucial for the acceleration of Ras-mediated OIS. These findings indicate that cellular senescence and SASP are evolutionally conserved in invertebrates and that studies in *Drosophila* could provide novel mechanistic insights into these phenomena.

## Regulation of cell cycle arrest in *Drosophila* senescent cells

DNA damage is known to be the major cause of cellular senescence [1, 51]. Studies in mammalian systems have indicated that Ras activation elicits DNA damage mainly through DNA hyper-replication [3, 10] and production of reactive oxygen species (ROS) [13, 51–55]. It has also been well established that the ROS-induced DNA damage triggers cellular senescence. Intriguingly, in *Drosophila* imaginal epithelium, Ras activation and dysfunction of the mitochondrial respiratory chain synergize in inducing ROS production and DNA damage [42, 43]. Ras^V12^/*mito* ^−/−^ cells show much larger amount of ROS production and DNA damage than Ras^V12^ cells or *mito* ^−/−^ cells. A recent study in human cell cultures has indicated that Ras^V12^ cells show elevated mitochondrial respiration via enhanced conversion of pyruvate to acetyl-CoA that is the origin of mitochondrial tricarboxylic acid (TCA) cycle [56]. Therefore, when the mitochondrial electron transport is downregulated in Ras^V12^ cells, large amounts of metabolic intermediates in mitochondrial respiration may be accumulated in mitochondria, which could affect ROS production.

It has been shown in mammals that DNA damage triggers cell cycle arrest and thereby induces cellular senescence [1, 51]. Upon DNA damage, p53 and p16 are upregulated [57–61] and thereby activating the p53/p21/Rb pathway [35, 36, 62, 63] and the p16/Rb pathway [62, 64]. DNA damage stabilizes p53 protein by repressing the ubiquitin ligase Mdm2 [57–59]. p53 directly activates transcription of p21 [35]. Both p21 and p16 positively regulate the function of retinoblastoma 1 (Rb1), a cell cycle keeper, by repressing the activities of CDKs. p21 represses the activity of the Cyclin E-CDK2 complex, while p16 represses the activity of the Cyclin D-CDK4-CDK6 complex, leading to the induction of cell cycle arrest. Intriguingly, the mechanism regulating expression of Cdk inhibitors during cellular senescence in *Drosophila* seems to be distinct from mammals in three ways. First, DNA damage is not involved in stabilization of *Drosophila* p53 (dp53) protein [42, 65]. *Drosophila* Ras^V12^/*mito* ^−/−^ cells, in which huge amount of ROS production and DNA damage occur, present larger elevation of dp53 than Ras^V12^ cells or *mito* ^−/−^ cells [42]. Nonetheless, this dp53 elevation is not blocked by suppression of ROS production, suggesting that dp53 protein level is not affected by oxidative DNA damage. Indeed, it has been reported that ionizing radiation (IR)-induced DNA damage does not change dp53 protein level, but it activates dp53 function via Loki (a Chk2 homologue)-dependent phosphorylation [65]. Similarly to mammalian Chk2, Loki acts as a kinase downstream of DNA damage-responsive kinases Tefu (an ATM homologue) and Mei-41 (an ATR homologue) [66, 67]. Thus, an alternative mechanism, not DNA damage, may stabilize dp53 protein, while DNA damage activates dp53 function. Second, dp53 does not regulate expression of *Drosophila* p21/p27, Dacapo [65, 68]. Loss of the dp53 gene in Ras^V12^/*mito* ^−/−^ cells does not block elevation of Dacapo (our unpublished data), which is consistent with previous reports indicating that dp53 does not participate in the regulation of Dacapo expression [65, 68]. Meanwhile, it has been shown that the expression level of Dacapo in Ras^V12^ cells is comparable with that in Ras^V12^/*mito* ^−/−^ cells but is much higher than that in *mito* ^−/−^ cells [42]. These observations indicate that Dacapo expression is dependent on Ras function but not dp53 function. In fact, previous studies have indicated that dp53 has a much closer relationship with apoptosis than cell cycle arrest [65, 69–72]. Finally, p16, another CDK inhibitor crucial for the induction of cellular senescence in mammals, is not conserved in *Drosophila*. Collectively, Ras^V12^-induced Dacapo elevation seems to be the central event triggering cell cycle arrest during cellular senescence in *Drosophila*.

The mechanism by which p53 regulates cyclin E protein stability, however, is conserved in *Drosophila*. It has been reported that dp53 induces ubiquitin-mediated proteolysis of cyclin E by activating gene expression of an E3 ubiquitin ligase Archipelago (Ago; a Fbxw7 homologue) [73–75]. It is known that gene transcription of mammalian Fbxw7 is positively regulated by p53 and that Fbxw7 leads to degradation of cyclin E through its ubiquitin ligase activity [76–78]. Together, these observations suggest that Ras^V12^-induced Dacapo upregulation and dp53-induced cyclin E degradation may cooperatively drive rigid cell cycle arrest in Ras^V12^/*mito* ^−/−^ cells in *Drosophila*.

## Roles of JNK and Hippo signaling in SASP

The c-Jun N-terminal kinase (JNK) pathway is a kinase cascade that mediates stress signaling such as oxidative stress and DNA damage [79–83]. *Drosophila* Ras^V12^/*mito* ^−/−^ senescent cells show much higher *Drosophila* JNK (dJNK; a JNK 1/2/3 homologue) activity than Ras^V12^ cells or *mito* ^−/−^ cells, and this dJNK activation is blocked by ROS inhibition [43]. Intriguingly, prominent activation of dJNK in Ras^V12^/*mito* ^−/−^ cells is achieved by cell cycle arrest [42]. Cyclin E overexpression in Ras^V12^/*mito* ^−/−^ cells inhibits dJNK activation without affecting ROS production [42]. In addition, Ras activation, which causes a weak induction of ROS, and loss of cyclin E synergistically trigger excessive activation of dJNK [42, 43]. Ras activation alone slightly increases dJNK activity, while loss of cyclin E alone is insufficient for the induction of dJNK activation. These observations suggest that cell cycle arrest can amplify dJNK activity without changing ROS level. Furthermore, dJNK activation can induce cell cycle arrest [42], which is consistent with a previous report showing that JNK1 stabilizes p21 protein via phosphorylation in a human colon cancer cell line [84]. Taken together, these data suggest the existence of a positive feedback loop between dJNK signaling and cell cycle arrest in Ras^V12^/*mito* ^−/−^ cells, and this loop and oxidative DNA damage may act synergistically to induce excessive activation of dJNK.

Previous reports have suggested a close link between JNK signaling and SASP. SASP is considered to be regulated by NF-κB signaling and epigenetic mechanisms in mammals. NF-κB signaling positively regulates SASP during cellular senescence downstream of Ras signaling [85–89]. Epigenetic mechanisms, such as chromatin remodeling, histone modification, and microRNA, also affect SASP [30, 90–94]. On the other hand, JNK has been shown to regulate expression of SASP factors including matrix remodeling factors and inflammatory cytokines both in mammals and *Drosophila*. As for matrix remodeling factors, mammalian JNK induces expression of Mmps via transcription factor activator protein-1 (AP-1) family [95–100], while dJNK induces Mmp1 elevation via *Drosophila* Fos (dFos), an AP-1 family member [101–103]. As for inflammatory cytokines, mammalian JNK induces elevation of IL-6 [104–106], IL-8 [107, 108], and monocyte chemoattractant protein-1 (MCP-1) [109–111], while dJNK induces elevation of Upd (an IL-6 homologue) [101, 112, 113]. In *Drosophila* Ras^V12^/*mito* ^−/−^ cells, dJNK upregulates Upd via inactivation of the Hippo pathway [42, 43]. The Hippo pathway is an evolutionally conserved tumor suppressor signaling that regulates cell proliferation and cell death [114, 115]. In mammals, Mst1/2 and Lats1/2, the core components of the Hippo pathway, repress the Hippo effectors Yap1/2 and Taz via phosphorylation [114, 116–120]. Similarly, in *Drosophila*, Hippo (a Mst1/2 homologue) and Warts (a Lats1/2 homologue) inactivate Yorkie (Yki; a Yap1 homologue) via phosphorylation [114, 116, 120–124]. Recent studies have reported that the Hippo pathway negatively regulates expression of SASP factors including IL-6 in mammals [125–128], similarly to *Drosophila* cells [129–132]. Marked upregulation of Upd in *Drosophila* Ras^V12^/*mito* ^−/−^ cells is blocked by expression of a dominant negative form of dJNK, cyclin E, Warts, or RNAi-mediated knockdown of Yki [42, 43]. Furthermore, it has been shown that dJNK signaling and Ras signaling cooperatively inactivate the Hippo pathway, thereby inducing SASP. Recent studies in *Drosophila* and human cell cultures have shown that JNK signaling and Ras signaling act synergistically to inhibit the Hippo pathway via Ajuba LIM protein (Jub)/Ajuba family proteins, which are known as Warts/LATS inhibitors [133–138]. Thus, Jub/Ajuba family proteins may also act as key regulators of SASP during cellular senescence. These findings indicate the importance of JNK signaling in the induction of SASP.

## Senescence or apoptosis?

Apart from cellular senescence, apoptosis also acts as a major defense mechanism against tumorigenesis [139]. Apoptosis is an active cell death program executed by killer proteases called caspases [140–142]. Are there any functional relationships between cellular senescence and apoptosis? Studies in *Drosophila* have indicated that Ras signaling negatively regulates the function of the pro-apoptotic protein head involution defective (Hid) both transcriptionally and post-transcriptionally, thereby suppressing apoptosis [143, 144]. Interestingly, senescent Ras^V12^/*mito* ^−/−^ cells seem to exhibit apoptosis resistance [42, 43]. On the other hand, in mammals, Ras signaling not only induces cellular senescence but also suppresses apoptosis [145, 146]. Interestingly, it has also been shown in mammals that senescent cells have the resistance to apoptosis [147–150]. Conversely, apoptosis inhibition by the pan-caspase inhibitor accelerates the anticancer agent-induced senescence in human culture cells, suggesting that apoptotic signaling antagonizes cellular senescence [151]. Therefore, two major tumor-suppressive machineries, cellular senescence and apoptosis, seem to counteract each other. Future studies on common signaling involved in both cellular senescence and apoptosis would increase our understanding of how these machineries cooperatively regulate tumorigenesis.

## Conclusions

**Fig. 2:**
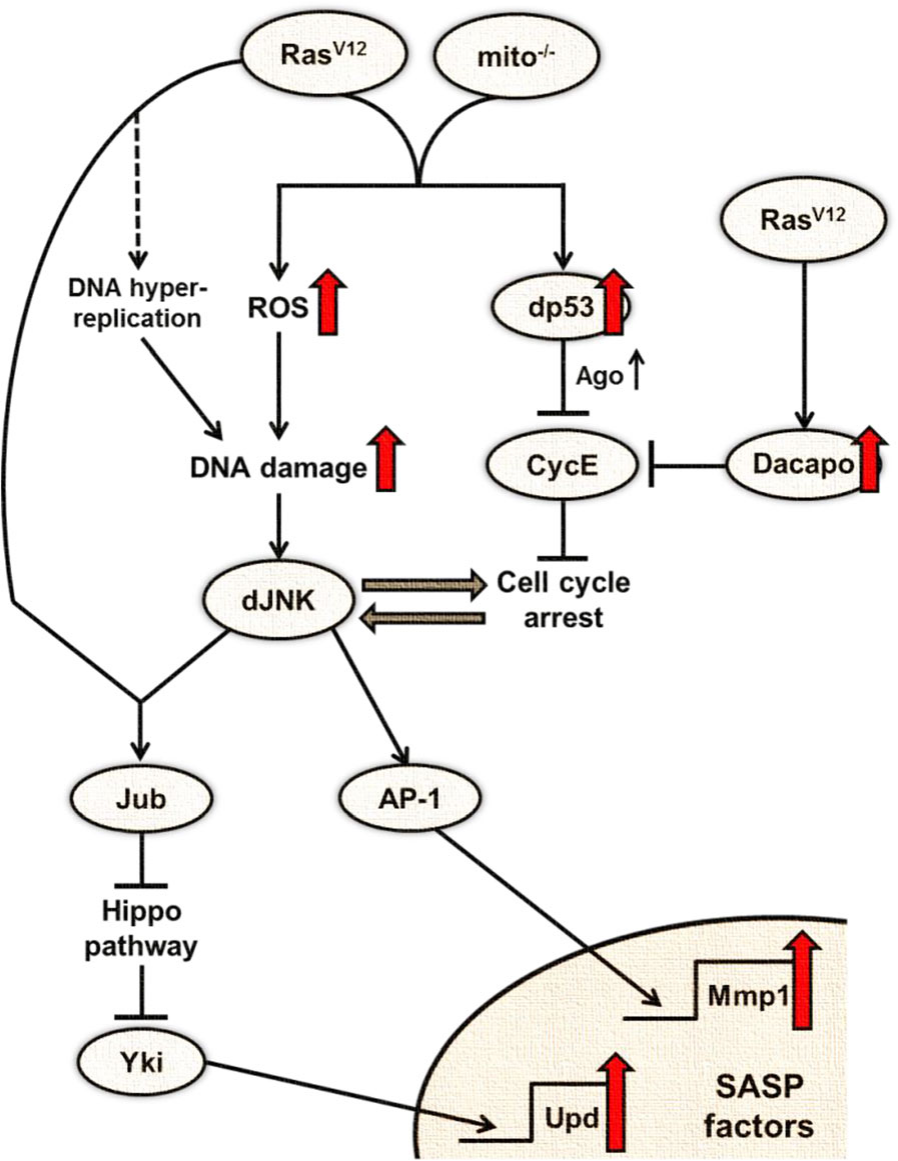
Scheme of the underlying mechanisms driving cellular senescence and SASP in *Drosophila* Ras^V12^/*mito* ^−/−^ cells

Recent studies in *Drosophila* have revealed that cellular senescence and SASP exist in invertebrates and that Ras activation and mitochondrial dysfunction synergistically drive cellular senescence and SASP via complex mechanisms mediated by JNK and Hippo signaling (Fig. 2). These findings have opened a new direction of the research field of cellular senescence. Future studies taking advantages of the powerful genetics of *Drosophila* would provide novel insights into cellular senescence and SASP, as well as new therapeutic strategies against cancers.

## Abbreviations

Ago: Archipelago
AP-1: Activator protein-1
CDK: Cyclin-dependent kinase
dFos: *Drosophila* Fos
dJNK: *Drosophila* JNK
dp53: *Drosophila* p53
Hid: Head involution defective
IL-6: Interleukin 6
IR: Ionizing radiation
JNK: c-Jun N-terminal kinase
Mmp: Matrix metalloprotease
OIS: Oncogene-induced senescence
Rb1: Retinoblastoma 1
ROS: Reactive oxygen species
SAHF: Senescence-associated heterochromatic foci
SASP: Senescence-associated secretory phenotype
SA-β-gal: Senescence-associated β-galactosidase
TCA: Tricarboxylic acid
Upd: Unpaired
Yki: Yorkie
